# 1827. Comparison of clinical outcomes for glycopeptides and beta-lactams in methicillin-susceptible *Staphylococcus aureus* bloodstream infections

**DOI:** 10.1093/ofid/ofac492.1457

**Published:** 2022-12-15

**Authors:** Yeon Ju La, Hye Rim Kim, Dong Hyun Oh, Jin Young Ahn, Yong Chan Kim

**Affiliations:** Division of Infectious disease, Department of Internal Medicine, Kangwon National University Hospital, Chuncheon, Republic of Korea, Chuncheon, Kangwon-do, Republic of Korea; Biostatistics Collaboration Unit, Department of Biomedical Systems Informatics, Yonsei University College of Medicine, Seoul, Republic of Korea, seoul, Seoul-t'ukpyolsi, Republic of Korea; Department of Internal Medicine, Seoul Medical Center, Seoul, Republic of Korea, seoul, Seoul-t'ukpyolsi, Republic of Korea; Yonsei University College of Medicine, seoul, Seoul-t'ukpyolsi, Republic of Korea; Department of Internal Medicine, Division of Infectious disease, Yongin Severance Hospital, Yonsei University College of Medicine, Yongin, Kyonggi-do, Republic of Korea

## Abstract

**Background:**

Several studies demonstrated the inferiority of glycopeptides as definitive antibiotics for MSSA BSI compared to anti-staphylococcal beta-lactam antibiotics. However, almost all of them were retrospective observational studies and no randomized controlled. Therefore, there remains the problem of bias in treatment selection and the possibility of residual confounding factors. In this study, we compare the therapeutic effects of glycopeptides and anti-staphylococcal beta-lactams in the treatment of MSSA BSI using inverse probability of treatment weighting (IPTW) analysis.

**Methods:**

This is a retrospective cohort study performed in double-center from January 1, 2010 to December 31, 2018. Patients (age ≥18 years) with MSSA identified in the blood culture were included. Patients were classified into two groups according to definite antibiotics used, the beta-lactam (nafcillin or cefazolin) group and the glycopeptide (vancomycin or teicoplanin) group.

**Figure 1.**
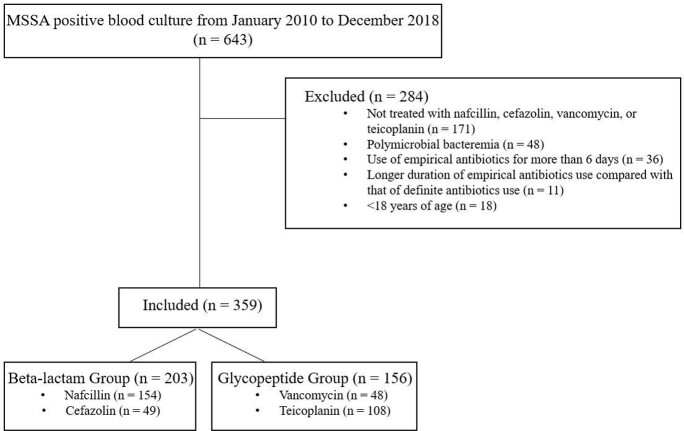
Flow chart of the study population

**Results:**

During the study period, 643 patients had MSSA bacteremia. Among them, 203 were treated with beta-lactam group and 156 were treated with glycopeptide group as definite therapy. (Figure 1). Comparison of clinical characteristics of patients between the beta-lactam and glycopeptide groups are shown in Table 1. After IPTW, baseline characteristics of the two groups were well balanced except for the primary focus of bacteremia. Although persistent bacteremia was less common in glycopeptide group (17.9% vs 4.0%; OR, 0.28; 95% CI, 0.14 – 0.60; *P* < 0.001), the glycopeptide group had higher overall mortality rate (15.9% vs 39.6%, *P* = 0.024), 7-day mortality rate (2.1% vs 14.1%, *P* < 0.001), and 28-day mortality rate (7.7% vs 30.9%, *P* = 0.012) (Table 2) than those of the beta-lactam group. When IPTW was augmented by multivariable logistic regression analyses to minimize remnant confounding, glycopeptide use for the treatment of MSSA BSI was associated with significant risk for 28-day mortality (adjusted OR, 3.37; 95% CI, 1.71–6.61; *P* < 0.001) (Figure 2).

**Figure:**
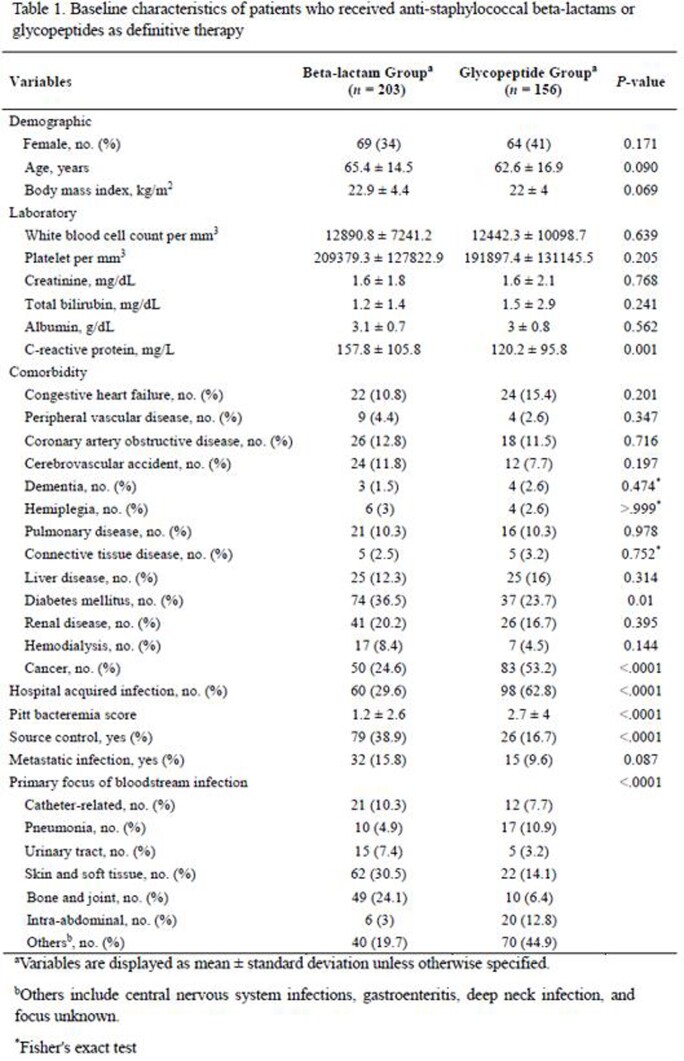

**Figure:**
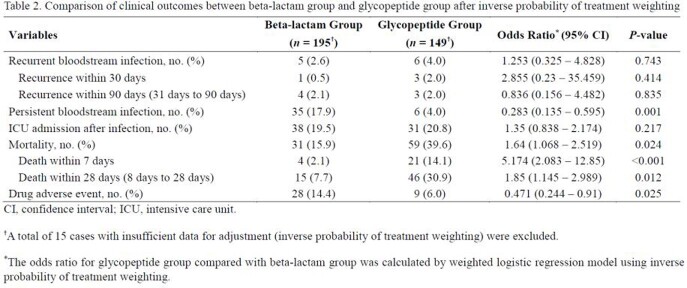

**Figure 2.**
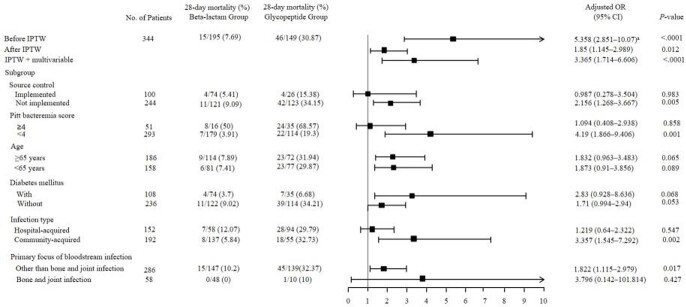
Adjusted ORs and 95% CIs for the primary end point in main analysis and various subgroups

Abbreviations: CI, confidence interval; OR, odds ratio; IPTW, inverse probability of treatment weighting.

^a^Unadjusted OR

**Conclusion:**

Definitive therapy with beta-lactams in patients with MSSA BSI was associated with lower 28-day mortality when compared with definitive therapy with glycopeptides. This study provides compelling evidence of anti-staphylococcal beta-lactam use for MSSA BSI treatment.

**Disclosures:**

**All Authors**: No reported disclosures.

